# Early-Life Environmental Determinants of Allergic Conditions in Children with Atopic Heredity: A Single Center Cross-Sectional Study from Bulgaria

**DOI:** 10.3390/medsci13030198

**Published:** 2025-09-18

**Authors:** Antoniya Hachmeriyan, Albena Toneva, Miglena Marinova-Achkar, Rouzha Pancheva

**Affiliations:** 1 Department of Physiology and Pathophysiology, Medical University Varna, 9002 Varna, Bulgaria; 2 NutriLect Research Group, Department of Neurosciences, Research Institute, Medical University Varna, 9002 Varna, Bulgaria; albena_t@abv.bg (A.T.); mig_mar@abv.bg (M.M.-A.); ruja.pancheva@mu-varna.bg (R.P.); 3 Department of Hygiene and Epidemiology, Medical University Varna, 9002 Varna, Bulgaria

**Keywords:** allergy, food allergy, atopic dermatitis, early-life exposures, maternal stress, formula feeding, infections, children, atopy

## Abstract

Background: Allergic diseases in early childhood are influenced by genetic predisposition and modifiable early-life exposures, including epigenetic mechanisms. Understanding the interplay between environmental factors and allergy development in children with atopic heredity is critical for prevention strategies. Objective: To investigate the associations between selected early-life environmental exposures and the development of allergic conditions in children with a positive family history of atopy. Methods: A cross-sectional study was conducted among 120 children aged 2 years (±5 months) with atopic heredity, recruited at the Medical University of Varna, Bulgaria (2017–2020). Data on sociodemographic background, prenatal exposures, birth mode, feeding practices, pet contact, daycare attendance, and infectious burden were collected via structured questionnaires and medical records. Allergic outcomes (food allergy and atopic dermatitis) were physician-confirmed. Statistical analyses included t-tests and chi-square tests. Results: Food allergy was diagnosed in 23.3% and atopic dermatitis in 21.7% of participants. Formula feeding was significantly more common in children with food allergy (66.7% vs. 38.1%; *p* = 0.020). A lower maternal pregnancy experience score was significantly associated with both food allergy (*p* = 0.021) and overall allergic outcomes (*p* = 0.004). Indoor smoking was more common in households of non-allergic children (*p* = 0.034). Children with food allergy had significantly more rhinopharyngitis episodes (*p* = 0.014) and longer infection duration. Higher gastroenteritis frequency and hospitalization rates were also noted in food-allergic children. Conclusions: In children with atopic heredity, early formula feeding, prenatal maternal stress, and infection burden were associated with increased risk of allergic conditions. This study underscores the importance of early-life psychosocial and environmental influences, possibly mediated by epigenetic mechanisms, in the development of childhood allergies. These findings highlight novel targets for early prevention and warrant further longitudinal research.

## 1. Introduction

Allergic diseases—including asthma, allergic rhinitis, food allergy, and atopic dermatitis—are among the most prevalent chronic conditions in childhood worldwide. Recent research highlights a central role for early-life environmental factors in their pathogenesis [1].

Food allergy is a frequent early feature of the atopic march, with a prevalence of 6–8% in early childhood and up to 40% among children with atopic dermatitis (AD) [2,3,4,5,6,7]. Risk is highest in moderate-to-severe AD, in which impaired skin-barrier function facilitates immune sensitization to food antigens [4,7,8]. Common allergens include cow’s milk, egg, peanut, wheat, soy, tree nuts, and fish, with age-dependent profiles [6,7,8,9]. These observations underscore the need to interrogate early-life factors that modify food-allergy risk in genetically predisposed children.

Environmental exposures during critical developmental windows can durably influence diseases’ programming. Prenatal exposure to air pollutants, tobacco smoke, pet allergens, delivery mode, feeding practices, and family structure has been associated with pathogenic marks linked to allergic disease, potentially producing sustained changes in immune development and function [10,11,12].

Maternal smoking in pregnancy has been associated with altered offspring DNA methylation and increased risks of asthma and allergic rhinitis. Nicotine permeates the placental barrier and exerts deleterious effects on the developmental processes of the fetal pulmonary and immune systems. Nicotine exposure can modify lung structure, disrupt normal cytokine signaling, and skew the immune system towards a Th2-dominated response, characteristic of allergic disease [13]. Smoke exposure in utero has also been linked to methylation changes in genes governing Th1/Th2 balance and immune regulation, with potential long-term effects on immune function and susceptibility to allergic diseases [14].

Mode of delivery has likewise been implicated. Infants born by Cesarean section (C-section) bypass exposure to maternal vaginal microbiota that contribute to immune education. Vaginally delivered infants typically acquire Bifidobacterium and Lactobacillus species that support balanced Th1/Th2 responses, whereas C-section neonates are more often colonized by skin or hospital flora, with delayed intestinal colonization and a potential Th2 bias associated with asthma, eczema, and food allergy [12,15,16,17].

Evidence on breastfeeding and allergic disease is mixed. Some studies suggest protection against AD, whereas others report null or even increased risk [18]. Nevertheless, international scientific societies recommend exclusive breastfeeding for at least 4–6 months to support primary prevention of allergic disease, promote a diverse microbiome, and aid immune maturation [19,20,21,22,23].

Pet exposure remains debated. Cats and dogs are common sources of household allergens that disseminate widely via dander, saliva, and hair, adhering to clothing and furnishings and reaching environments without pets [24,25]. However, the effects of this exposure seem to vary depending on several factors, including the timing of exposure, the type of pet, and the genetic predisposition of the child.

The primary objective of this study was to investigate associations between selected early-life exposures and the development of allergic disease in young children with a documented familial predisposition to atopy. We focused on early-life exposures (maternal stress, feeding type, infections, smoking, delivery mode) that have been described in the literature as potential modifiers of immune development. Although we did not measure epigenetic biomarkers directly, we conceptualized these exposures as “epigenetically influenced” determinants based on prior evidence of effects on DNA methylation and immune regulation.

## 2. Materials and Methods

### 2.1. Study Design and Setting

We conducted a cross-sectional study at the Medical University “Prof. Dr. Paraskev Stoyanov,” Varna, Bulgaria (2017–2020). Pregnant women were recruited if atopic heredity was present, defined as ≥1 first-degree relative (parent or sibling) with a physician-diagnosed allergic disease. Of 1210 eligible women approached, 156 consented; 120 mother–child dyads were retained for analysis after excluding 36 for incomplete data or withdrawal. Participation was voluntary and may have introduced selection bias (e.g., greater engagement among health-conscious families); this is considered in interpreting the findings. No a priori sample-size calculation was performed given the exploratory nature of this study.

Ethics approval was granted by the Ethics Committee for Research at the Medical University “Prof. Dr. P. Stoyanov”—Varna (Protocols No. 60 and No. 91, date of approval 23 February 2017 and 21 February 2020).Written informed consent was obtained from all parents or legal guardians. Confidentiality and data protection were strictly maintained.

### 2.2. Participants and Inclusion Criteria

Eligible children were aged 2 years (±5 months), born at term (≥37 weeks’ gestation) with birth weight ≥ 2500 g, and had a positive family history of allergic disease in the mother, father, or a sibling. Exclusion criteria were preterm birth, perinatal complications (e.g., asphyxia, trauma), congenital anomalies, genetic disorders, or chronic comorbidities. Eligible participants were children aged 2 years (±5 months), born at term (≥37 weeks’ gestation) with birth weights ≥ 2500 g, and with a positive family history of allergic diseases in the mother, father, or sibling. Exclusion criteria included preterm birth, perinatal complications (e.g., asphyxia, trauma), congenital anomalies, genetic disorders, and chronic comorbid conditions.

### 2.3. Data Collection

After obtaining informed consent, caregivers completed a comprehensive structured questionnaire. Each child underwent a screening assessment to confirm eligibility.

### 2.4. Measures

Sociodemographic variables: child age, sex, and ethnicity; parental ages and education; family size; and urban vs. rural residence.

Medical history and clinical outcomes.

Pregnancy experience was assessed using a self-reported 10-point Likert-type scale (1 = very negative; 10 = very positive). Although not validated, this measure was intended to capture subjective pregnancy experience.

Allergic outcomes by age 2 included physician-diagnosed AD, allergic rhinoconjunctivitis, food allergy, urticaria, or asthma, confirmed via standardized questionnaires and medical records.

Acute illnesses included type and frequency of non-allergic conditions (respiratory, gastrointestinal, dermatological, etc.) during follow-up. We did not apply standardized severity scores for respiratory infections; burden was assessed by frequency, episode duration, and hospitalizations.

### 2.5. Environmental Exposures

Parental smoking. Maternal smoking during pregnancy (presence and cigarettes/day), paternal smoking status, and household tobacco-smoke exposure.

Animal contact. Household pet exposure during the first two years of life.

Early feeding. Feeding mode in early infancy (exclusive breastfeeding, mixed, or exclusive formula) and total breastfeeding duration (parent-reported).

### 2.6. Data Management and Statistical Analysis

Data were anonymized and entered into a secure, password-protected database with double-entry verification. Analyses were conducted in jamovi v2.2.2. Descriptive statistics summarized demographic, environmental, and clinical characteristics. Normality was assessed with Shapiro–Wilk tests and visual inspection. Because distributions approximated normality, we used parametric tests (independent-samples t test); sensitivity analyses with Mann–Whitney U tests were comparable. Continuous variables are reported as mean (SD); categorical variables as *n* (%). Group comparisons used t tests for continuous data and χ^2^ or Fisher’s exact tests for categorical data, as appropriate. Given the exploratory design, no formal multiple-comparison correction was applied; results should be interpreted cautiously. Two-sided tests were used, with statistical significance at *p* < 0.05.

## 3. Results

### 3.1. Sociodemographic and Perinatal Characteristics Associated with Allergy Status

Among 120 children, 28 (23.3%) had food allergy and 26 (21.7%) had AD by age two. Most demographic and perinatal comparisons were not statistically significant (Table 1A,B). Maternal and paternal age, parental education, ethnicity, and urban residence (>90% overall) did not differ by allergy status. Birth weight and sex distribution were similar across groups, although boys were more frequent among children with AD (75.0% vs. 51.9%). Exploratory sex-stratified analyses did not show consistent or significant effects; this study was underpowered for definitive subgroup analyses.

### 3.2. Early-Life Environmental Exposures and Allergy Risk

Key associations are summarized in Table 2, Table 3, Table 4 and Table 5. Mothers of children with food allergy reported significantly lower pregnancy-experience scores than mothers of non-allergic children (6.73 ± 2.76 vs. 8.09 ± 1.99; *p* = 0.021). The trend persisted when comparing any allergy vs. none (7.26 vs. 8.39; *p* = 0.004).

C-section was more common among children with food allergy (60.0% vs. 49.5%) and less common among those with AD (43.8% vs. 51.9%), but differences were not significant. First-born children were proportionally more frequent in allergic groups (food allergy, 60.0%; AD, 62.5%) than in non-allergic groups (45.7% and 45.2%, respectively).

Formula feeding in infancy was significantly more common among children with food allergy (66.7% vs. 38.1%; *p* = 0.020) (Table 3A). Breastfeeding at birth was more frequent in children with food allergy (100% vs. 79%; *p* = 0.050), although sustained breastfeeding did not differ by outcome.

Trends suggested higher pet exposure among children with food allergy and lower exposure among those with AD, but differences were not significant. Daycare attendance, parental smoking, and delivery mode showed no significant associations with allergic outcomes. Notably, indoor smoking was reported only in households of non-allergic children (12.9% vs. 0%; *p* = 0.034), which may reflect behavior modification in families perceiving higher atopic risk (Table 4A,B).

### 3.3. Infectious Burden in Relation to Allergy Status

Respiratory infections comprised the majority of illnesses (Table 5A,B). Children with food allergy had more respiratory episodes than non-allergic peers (5.20 ± 5.83 vs. 3.10 ± 3.62; *p* = 0.056), and the association was significant when comparing any allergy vs. none (4.24 ± 5.34 vs. 2.73 ± 2.51; *p* = 0.041). Rhinopharyngitis was more frequent in children with food allergy (3.80 ± 4.87 vs. 1.95 ± 2.22; *p* = 0.014).

Infections also tended to last longer in allergic children. Duration of respiratory infections was borderline longer in those with any allergy (34.32 ± 46.53 vs. 21.63 ± 21.10 days; *p* = 0.046). Rhinopharyngitis episodes lasted longer in children with food allergy (27.93 ± 37.87 vs. 12.78 ± 15.91 days; *p* = 0.007). Children with food allergy had more acute gastroenteritis (0.87 ± 1.19 vs. 0.31 ± 0.75; *p* = 0.016) with longer duration (3.67 ± 4.67 vs. 1.44 ± 3.37 days; *p* = 0.025) and were more often hospitalized for gastroenteritis (0.27 ± 0.59 vs. 0.06 ± 0.27; *p* = 0.022). Overall hospitalization rates were low and did not differ significantly by group.

## 4. Discussion

### 4.1. Pregnancy Experience

Maternal stress in pregnancy is increasingly recognized as a modifiable risk factor for childhood allergic disease (especially asthma, eczema, and wheeze). we observed significantly lower pregnancy-experience scores among mothers of children with food allergy, suggesting a psychobiological factor that has not been widely examined as an independent exposure. prior studies report dose–response relationships, with multiple or severe stressors increasing allergic risk and possible sex-specific susceptibility [26,27,28,29]. Proposed mechanisms include HPA-axis activation with elevated cortisol crossing the placenta, placental dysfunction, and microbiome perturbations [30,31]. null findings have also been reported, with no significant association between maternal stressful life events and atopic dermatitis in children aged 4–6, likely reflecting heterogeneity in stress definitions, timing, outcomes, and confounding control [29].

### 4.2. Maternal Smoking

Extensive evidence links maternal smoking (and prenatal passive exposure) to increased risks of wheeze and asthma in offspring, including when postnatal smoke exposure is absent [32,33]. Observational data suggest dose–response relationships with cigarettes per day and trimester-specific effects, and these associations often persist after adjustment for birth weight and other perinatal factors, implying mechanisms beyond fetal growth restriction [32,33]. In utero smoke exposure has also been associated with greater sensitization to inhalant allergens, particularly in children of atopic mothers, though links to food sensitization are less consistent and align with our findings [14]. Proposed biological pathways include oxidative stress and systemic inflammation, placental dysfunction, impaired alveolar and airway development, and epigenetic remodeling of immune-regulatory mechanisms that may skew T-cell activity and mucosal immunity [34,35,36]. Some studies further indicate sex-specific vulnerability and potential interactions with familial atopy, consistent with immune programming during critical windows [32,33,34,35,36].

In our cohort, smoking variables were not significantly associated with allergic outcomes; notably, indoor smoking was paradoxically less common in allergic households. We interpret this pattern as likely behavioral (risk-avoidant changes after early symptoms or diagnosis) rather than biological. Misclassification from self-report, low exposure prevalence, and limited power for subgroup analyses could also bias estimates toward the null. Future longitudinal work with objective exposure markers and epigenetic readouts may clarify temporality and mechanisms in at-risk populations.

### 4.3. C-Section

Our data suggested non-significant trends between C-section and both food allergy and AD. The literature indicates higher risk of food allergy (notably cow’s-milk allergy) after C-section, with stronger associations in children with parental atopy, whereas associations with AD are generally weak or inconsistent [15,37,38]. Several cohorts report higher risks of wheeze/asthma and atopic sensitization after C-section, particularly in atopic families [39,40], although large studies have found null associations after adjustment for confounders [41]. A recent meta-analysis reported a modest but significant association with asthma, varying by sex and region [42]. In addition to the maternal microflora exposure, vaginal birth triggers stress-related hormonal changes in newborns, which may aid immune system maturation. Cesarean section, via altered early-life microbial exposures and downstream effects on immune regulation, has been correlated with heightened risk of food allergy in offspring, while evidence regarding atopic dermatitis is less definitive.

### 4.4. Early Feeding Practice

Consistent with our data, formula feeding was more frequent among children who developed food allergy. This pattern may reflect both causal and non-causal pathways. Biologically, the absence of human-milk immunologic constituents—such as secretory IgA, human milk oligosaccharides, and tolerogenic cytokines—could influence early microbial colonization and oral-tolerance induction, plausibly increasing the likelihood of IgE-mediated responses in genetically susceptible infants [2,3,4,5,6,7]. At the same time, reverse causation is possible: early eczematous symptoms, feeding difficulties, or parental concern about allergy may prompt earlier or predominant formula use (including hypoallergenic preparations), which can confound observed associations [43].

Infants with AD and atopic heredity are at increased risk of IgE-mediated food allergy, while asymptomatic sensitization is common; therefore, indiscriminate IgE testing and elimination diets should be avoided without objective confirmation (e.g., oral food challenge) to prevent nutritional harm [44]. Preventive strategies increasingly emphasize timely oral exposure to allergenic foods—particularly peanut—in the context of developmental readiness and appropriate supervision, alongside optimized emollient use to support the skin barrier in AD [9,10,43,45]. Implementation details (age at introduction, dosing, and maintenance exposure) and adherence likely modify effectiveness and may account for some heterogeneity across cohorts [46].

Evidence regarding breastfeeding and AD remains heterogeneous, with studies showing protection, no effect, or context-specific benefits; variability in definitions (exclusive vs. any breastfeeding), duration and timing, maternal atopy, recall bias, and outcome ascertainment likely contribute to inconsistency [47,48]. Rigorous randomized trials and mechanistic studies are needed to disentangle causal effects from confounding and to clarify how breastfeeding, early allergen introduction, and barrier-supportive care interact to shape allergic risk trajectories [49,50].

### 4.5. Sibings

First-born status was more common among allergic children (≈60%), consistent with literature showing lower atopy prevalence with increasing sibship size. Beyond simple counts, both birth order and age spacing appear relevant: protection is typically strongest with the presence of older siblings and with close inter-sibling contact in early life, when immune education is most plastic [51,52]. Proposed mechanisms center on enhanced microbial sharing within larger households (respiratory and enteric viruses, skin and oral commensals, endotoxin), which may bias immune maturation away from Th2-skewed responses toward more balanced Th1/Th17 pathways and promote regulatory T-cell development [53,54]. Residual confounding—by daycare use, pet ownership, parental smoking, and socioeconomic factors—remains a concern, but the overall pattern across cohorts and meta-analyses suggests a modest, reproducible protective effect of sibship, particularly for sensitization and wheeze phenotypes [55].

### 4.6. Pets

Our study showed some non-conclusive but mixed trends towards pet exposure and its link with food allergy (40% vs. 22.9%) and atopic dermatitis (6.3% vs. 27.9%). Prior studies are heterogeneous, with species-, timing-, and dose-dependent effects. Early dog exposure is variably associated with reduced atopic sensitization and possibly lower risks of asthma and food allergy, potentially via higher endotoxin loads and distinct microbiota that augment microbial diversity. Cat exposure more often increases sensitization, likely reflecting persistent airborne allergens with high environmental burden [56,57,58].

Studies on early pet exposure show mixed results regarding allergy and asthma development. Some research suggests early exposure- especially to dog- may protect against food allergies and allergic sensitization, while other studies link pet ownership to increased respiratory allergies, indicating the effect may depend on genetics and environment [59]. The “hygiene hypothesis” proposes that early exposure to microbes, such as those from pets, supports immune system development and reduces the risk of atopic diseases. Supporting this, children exposed to dogs or cats in their first year had a lower risk of allergic sensitization, with a stronger effect seen when multiple pets were present [60]. Early dog exposure reduced the risk of sensitization to airborne allergens [59]. Other studies also link dog ownership to lower risks of asthma and food allergies, possibly due to increased microbial exposure [61].

Effects may be modified by genetic susceptibility (e.g., atopic heredity, barrier dysfunction), window of exposure (prenatal vs. first year), and indoor intensity (sleeping areas, carpeting, cleaning practices) [62]. Interpretation is further complicated by avoidance bias (families with parental atopy may choose not to keep pets) and by reverse causation after early symptoms. Literature showed contradictory results: some studies report protective effects, others increased risks. Current study’s mixed findings are aligned with this ambiguity.

### 4.7. Daycare Attendance

A total of 46.7% of the participants attended daycare. Evidence remains mixed: some cohorts indicate that early entry (often <6–12 months) is associated with lower subsequent asthma risk, consistent with the hygiene hypothesis, while simultaneously increasing early-life wheezing and infections that do not invariably track to persistent asthma at school age [63,64,65]. Some researchers found an inverse relationship between early daycare attendance and the prevalence of rhinoconjunctivitis, suggesting that daycare attendance might protect against some allergic conditions by promoting immune system maturation through exposure to various pathogens [64]. For eczema, results are inconsistent—ranging from no association to context-specific protection- likely reflecting differences in exposure intensity, infection profiles, and household microbial background [63,66,67]. Limited data suggest that daycare, by broadening microbial diversity and oral exposure, could reduce the risk of food allergy in some settings, but confounding by selection into daycare (e.g., parents of symptomatic infants delaying enrollment) and by antibiotic use remains substantial [68]. The underlying mechanism relates to the hygiene hypothesis, which suggests that increased microbial exposure in communal environments fosters immune tolerance. By interacting with a diverse range of microbes, infants in daycare may develop a more robust immune response, reducing the likelihood of food sensitization. This effect parallels findings in studies on respiratory allergies and eczema, where greater microbial diversity is linked to lower allergic disease rates. Overall, daycare appears to function as a complex proxy for early microbial and social exposures, with effects that depend on timing, duration, and co-exposures.

### 4.8. Infectious Burden

We identified a higher frequency and longer duration of several infections among allergic children, particularly those with food allergy, including more respiratory infections and rhinopharyngitis, as well as more frequent and prolonged gastroenteritis with higher hospitalization for gastroenteritis. These findings accord with reports of greater cutaneous and extracutaneous infection susceptibility in atopic individuals, potentially reflecting Th2-skewed immunity, attenuated type I/III interferon responses to respiratory viruses, and barrier dysfunction (e.g., increased transepidermal water loss and *S. aureus* colonization in AD) [69,70,71]. Bidirectionality is plausible: infections may exacerbate allergic inflammation, while allergic inflammation and barrier defects may facilitate infections and prolong recovery. Alternative explanations include healthcare-seeking differences, antibiotic exposure, and measurement limitations (self-report, lack of standardized severity scores). Future work incorporating pathogen-specific testing, objective severity metrics, and time-to-event analyses, with adjustment for daycare, siblings, and pet exposures, will be important to clarify temporality and mechanisms [72].

### 4.9. Limitations

This study has several limitations. (1). Its cross-sectional design precludes inference of causality between exposures and allergy outcomes. (2). The relatively small sample size limited statistical power, especially for subgroup analyses (e.g., sex differences). (3). The pregnancy experience score was based on a non-validated scale, which may limit reliability. (4). Follow-up only extended to age two, when many allergic conditions may not yet be fully expressed. Longer prospective studies with biomarker analysis are needed to confirm mechanistic pathways. (5). The single-center recruitment and modest participation rate raise the possibility of selection bias and limit generalizability. The absence of an a priori sample size calculation is also a limitation and may have affected statistical power, particularly for subgroup analyses. (6). This study lacked detailed clinical severity measures for respiratory infections; only duration and hospitalization rates were available as proxies. (7). Finally, while multiple comparisons were performed, no adjustment was made, as this was an exploratory study. These limitations should be considered when interpreting the findings, which remain hypothesis-generating and require confirmation in larger longitudinal studies.

## 5. Conclusions

Our results support the pivotal role of early-life environmental factors with possible epigenetic relevance in childhood allergic disease. Novelty in this study includes the psychological dimension, indicated by lower maternal pregnancy experience scores significantly associated with allergy outcomes; the unexpected finding related to indoor smoking, possibly indicative of altered behavioral practices in at-risk families, and the specific, quantitatively detailed relationship between allergy status and infectious burden (frequency and duration), particularly for respiratory and gastroenteric infections. These data refine current understanding and point to future longitudinal studies incorporating specific biomarkers and psychosocial measures.

## Figures and Tables

**Table 1:** (**A**). Sociodemographic characteristics by food allergy status (FA). (**B**). Sociodemographic characteristics by atopic dermatitis status (AD).

(A)			
Characteristic	Food Allergy (*n* = 28)	No Food Allergy (*n* = 92)	*p*-Value
Age at questionnaire (months)	24.73 ± 3.90	23.90 ± 3.88	0.441
Boys, *n* (%)	60.0	54.3	0.677
Mother’s age (years)	30.40 ± 4.58	30.61 ± 4.53	0.867
Father’s age (years)	31.27 ± 3.88	33.66 ± 4.76	0.066
Mother’s education (higher), *n* (%)	53.3	66.7	0.192
Father’s education (higher), *n* (%)	40.0	42.9	0.601
Ethnicity (Bulgarian), *n* (%)	86.7	91.4	0.521
Urban living, *n* (%)	93.3	96.2	0.604
(**B**)			
**Characteristic**	**Food Dermatitis (*n* = 26)**	**No Food Dermatitis (*n* = 94)**	***p*-Value**
Age at questionnaire (months)	24.94 ± 2.98	23.87 ± 3.99	0.305
Boys, *n* (%)	75.0	51.9	0.084
Mother’s age (years)	31.25 ± 4.17	30.48 ± 4.58	0.528
Father’s age (years)	33.88 ± 4.51	33.28 ± 4.76	0.639
Mother’s education (higher), *n* (%)	87.5	60.6	0.109
Father’s education (higher), *n* (%)	56.3	40.4	0.481
Ethnicity (Bulgarian), *n* (%)	93.8	90.4	0.045
Urban living, *n* (%)	100.0	95.2	0.370
Boys, *n* (%)	75.0	51.9	0.084

**Table 2:** (**A**). Prenatal and perinatal characteristics by food allergy status (FA); (**B**). Prenatal and perinatal characteristics by atopic dermatitis status (AD).

(A)			
Characteristic	FA (*n* = 28)	No FA (*n* = 92)	*p*-Value
Pregnancy experience (score)	6.73 ± 2.76	8.09 ± 1.99	0.021
Maternal smoking during pregnancy, *n* (%)	3 (20.0)	13 (12.4)	0.417
Cesarean delivery, *n* (%)	9 (60.0)	52 (49.5)	0.448
Birth weight (g)	3392.67 ± 355.42	3373.43 ± 395.06	0.859
(**B**)			
**Characteristic**	**AD (*n* = 28)**	**No AD (*n* = 94)**	***p*-Value**
Pregnancy experience (score)	7.25 ± 2.41	8.02 ± 2.09	0.182
Maternal smoking during pregnancy, *n* (%)	1 (6.3)	15 (14.4)	0.371
Cesarean delivery, *n* (%)	7 (43.8)	54 (51.9)	0.543
Birth weight (g)	3290.63 ± 310.15	3388.94 ± 399.37	0.349

**Table 3:** (**A**). Early feeding by food allergy status (FA); (**B**). Early feeding by atopic dermatitis status (AD).

(A)			
Characteristic	Food Allergy (*n* = 15 for BF Row)	No Food Allergy (*n* = 83 for BF Row)	*p*-Value
Breastfeeding at birth, *n* (%)	15 (100%)	83 (79%)	0.050
Formula milk (vs. other), *n* (%)	10 (66.7%)	40 (38.1%)	0.020
Feeding method (breastfeeding), *n* (%)	5 (33.3%)	29 (27.6%)	0.646
Weight-for-age z-score at 1 year, mean ± SD	0.62 ± 1.46	0.52 ± 1.37	0.791
(**B**)			
**Characteristic**	**Atopic Dermatitis (*n*** **= 16 for BF Row)**	**No Atopic Dermatitis (*n* = 104 for BF Row)**	***p*-Value**
Breastfeeding at birth, *n* (%)	15 (93.8%)	83 (79.8%)	0.180
Formula milk (vs. other), *n* (%)	6 (37.5%)	44 (42.3%)	0.301
Feeding method (breastfeeding), *n* (%)	7 (43.8%)	27 (26.0%)	0.142
Weight-for-age z-score at 1 year, mean ± SD	0.73 ± 1.29	0.51 ± 1.39	0.549

**Table 4:** (**A**). Postnatal factors by food allergy status (FA); (**B**) Postnatal factors by atopic dermatitis status (AD).

(A)			
Characteristic	Food Allergy	No Food Allergy	*p*-Value
Parental smoking, *n* (%)	8 (53.3%)	56 (53.3%)	1.000
Mother smoking, *n* (%)	4 (26.7%)	32 (30.5%)	0.763
Father smoking, *n* (%)	6 (40.0%)	46 (43.8%)	0.781
Indoor smoking, *n* (%)	0 (0%)	10 (9.5%)	0.212
Only child (1 child), *n* (%)	60.0	45.7	0.501
Daycare attendance, *n* (%)	7 (53.3%)	49 (53.3%)	1.000
Presence of pet, *n* (%)	6 (40.0%)	24 (22.9%)	0.151
(**B**)			
**Characteristic**	**Atopic Dermatitis**	**No Atopic Dermatitis**	***p*-Value**
Parental smoking, *n* (%)	6 (37.5%)	58 (55.8%)	0.173
Mother smoking, *n* (%)	2 (12.5%)	34 (32.7%)	0.101
Father smoking, *n* (%)	5 (31.3%)	47 (45.2%)	0.295
Indoor smoking, *n* (%)	0 (0%)	10 (9.6%)	0.195
Only child (1 child), *n* (%)	62.5	45.2	0.335
Daycare attendance, *n* (%)	10 (62.5%)	46 (44.2%)	0.173
Presence of pet, *n* (%)	1 (6.3%)	29 (27.9%)	0.063

**Table 5:** (**A**). Infection burden by food allergy status (FA); (**B**). Infection burden by atopic dermatitis status (AD).

(A)			
Characteristic	Food Allergy	No Food Allergy	*p*-Value
Total infections, mean ± SD	6.40 ± 6.02	4.08 ± 4.01	0.055
Respiratory infections (number), mean ± SD	5.20 ± 5.83	3.10 ± 3.62	0.056
Rhinopharyngitis (number), mean ± SD	3.80 ± 4.87	1.95 ± 2.22	0.014
Bronchiolitis (number), mean ± SD	1.13 ± 1.25	0.70 ± 2.08	0.429
Laryngitis (number), mean ± SD	0.27 ± 0.80	0.21 ± 0.65	0.756
Acute pneumonia (number), mean ± SD	0.13 ± 0.52	0.21 ± 0.62	0.649
Acute gastroenteritis (number), mean ± SD	0.87 ± 1.19	0.31 ± 0.75	0.016
Acute urinary infections (number), mean ± SD	0.00 ± 0.00	0.09 ± 0.44	0.455
Other infections (number), mean ± SD	0.20 ± 0.41	0.28 ± 0.85	0.734
Total duration of infections (days), mean ± SD	45.93 ± 43.35	30.57 ± 35.73	0.132
Respiratory infections (duration, days), mean ± SD	40.87 ± 42.95	24.92 ± 32.87	0.094
Rhinopharyngitis (duration, days), mean ± SD	27.93 ± 37.87	12.78 ± 15.91	0.007
Bronchiolitis (duration, days), mean ± SD	9.60 ± 11.41	7.03 ± 21.11	0.646
Laryngitis (duration, days), mean ± SD	1.33 ± 3.52	1.05 ± 3.07	0.741
Acute pneumonia (duration, days), mean ± SD	2.00 ± 7.75	3.43 ± 9.81	0.590
Acute gastroenteritis (duration, days), mean ± SD	3.67 ± 4.67	1.44 ± 3.37	0.025
Acute urinary infections (duration, days), mean ± SD	0.00 ± 0.00	1.78 ± 9.84	0.486
Number of hospitalizations, mean ± SD	0.53 ± 0.83	0.42 ± 1.06	0.691
Hospitalizations for respiratory infections, mean ± SD	0.27 ± 0.59	0.31 ± 0.88	0.840
Hospitalizations for gastroenteritis, mean ± SD	0.27 ± 0.59	0.06 ± 0.27	0.022
Hospitalizations for urinary infections, mean ± SD	0.00 ± 0.00	0.03 ± 0.17	0.511
(**B**)			
**Characteristic**	**Atopic Dermatitis**	**No Atopic Dermatitis**	***p*-Value**
Total infections, mean ± SD	4.73 ± 2.92	4.34 ± 4.56	0.745
Respiratory infections (number), mean ± SD	3.75 ± 2.98	3.30 ± 4.14	0.675
Rhinopharyngitis (number), mean ± SD	2.13 ± 2.58	2.19 ± 2.77	0.927
Bronchiolitis (number), mean ± SD	1.00 ± 0.89	0.71 ± 2.12	0.593
Laryngitis (number), mean ± SD	0.19 ± 0.40	0.22 ± 0.70	0.851
Acute pneumonia (number), mean ± SD	0.25 ± 0.58	0.19 ± 0.61	0.723
Acute gastroenteritis (number), mean ± SD	0.50 ± 0.89	0.37 ± 0.83	0.549
Acute urinary infections (number), mean ± SD	0.06 ± 0.25	0.08 ± 0.43	0.897
Other infections (number), mean ± SD	0.13 ± 0.34	0.29 ± 0.86	0.453
Total duration of infections (days), mean ± SD	36.69 ± 27.13	31.85 ± 38.27	0.627
Respiratory infections (duration, days), mean ± SD	31.94 ± 26.69	26.14 ± 35.58	0.534
Rhinopharyngitis (duration, days), mean ± SD	15.38 ± 20.33	14.57 ± 20.48	0.883
Bronchiolitis (duration, days), mean ± SD	8.06 ± 9.94	7.24 ± 21.31	0.880
Laryngitis (duration, days), mean ± SD	1.50 ± 3.29	1.02 ± 3.09	0.567
Acute pneumonia (duration, days), mean ± SD	3.75 ± 8.66	3.17 ± 9.73	0.823
Acute gastroenteritis (duration, days), mean ± SD	2.13 ± 4.06	1.65 ± 3.55	0.629
Acute urinary infections (duration, days), mean ± SD	1.88 ± 7.50	1.51 ± 9.48	0.883
Number of hospitalizations, mean ± SD	0.38 ± 0.72	0.44 ± 1.08	0.810
Hospitalizations for respiratory infections, mean ± SD	0.25 ± 0.58	0.32 ± 0.88	0.769
Hospitalizations for gastroenteritis, mean ± SD	0.13 ± 0.50	0.08 ± 0.30	0.593
Hospitalizations for urinary infections, mean ± SD	0.00 ± 0.00	0.03 ± 0.17	0.496

## Data Availability

The original contributions presented in this study are included in the article. Further inquiries can be directed to the corresponding author.
